# Dr. S. H. Warren

**Published:** 1903-12

**Authors:** 


Dr. S. H. Warren, a graduate of the University of Buffalo, 1880, died in this city, October 29, 1903.

